# Scaling up evidence-based digital early life nutrition interventions in a county setting: an implementation trial – protocol for Phase 2 of the *Nutrition Now* project

**DOI:** 10.3389/fpubh.2023.1326787

**Published:** 2024-01-09

**Authors:** Anine Christine Medin, Frøydis Nordgård Vik, Christine Helle, Sissel Heidi Helland, Andrew Keith Wills, Natalie Garzon Osorio, Henrik Lian, Torunn Iveland Ersfjord, Wim Van Daele, Tormod Bjørkkjær, Erlend Nuland Valen, Mekdes Kebede Gebremariam, Erik Grasaas, Charlotte Kiland, Ulrica von Thiele Schwarz, Marianne Hope Abel, Penny Love, Karen Campbell, Harry Rutter, Mary Elizabeth Barker, Elisabet Rudjord Hillesund, Nina Cecilie Øverby

**Affiliations:** ^1^Department of Nutrition and Public Health, Faculty of Health and Sport Sciences, University of Agder, Priority Research Centre Lifecourse Nutrition, Kristiansand, Norway; ^2^Department of Community Medicine and Global Health, Institute of Health and Society, University of Oslo, Oslo, Norway; ^3^Department of Political Science and Management, Faculty of Social Sciences, University of Agder, Kristiansand, Norway; ^4^School of Health, Care and Social Welfare, Mälardalen University, Västerås, Sweden; ^5^Procome, Medical Management Centre, LIME, Karolinska Institutet, Stockholm, Sweden; ^6^Centre for Evaluation of Public Health Measures, Norwegian Institute of Public Health, Oslo, Norway; ^7^Institute for Physical Activity and Nutrition, School of Exercise and Nutrition Science, Deakin University, Geelong, VIC, Australia; ^8^Department of Social and Policy Sciences, University of Bath, Bath, United Kingdom; ^9^School of Health Sciences, University of Southampton, Southampton, United Kingdom; ^10^MRC Lifecourse Epidemiology Centre, University of Southampton, Southampton General Hospital, Southampton, United Kingdom

**Keywords:** implementation, digital dietary intervention, early life, first 1,000 days, maternal and child health care, feeding practices, municipality scale up, early childhood education and care

## Abstract

**Background:**

Few effective health interventions transition from smaller efficacy or effectiveness studies to real-world implementation at scale, representing a gap between evidence and practice. Recognising this, we have developed *Nutrition Now* – a tailored digital resource building on four efficacious dietary interventions, aiming to improve nutrition in the important first 1,000 days of life. *Nutrition Now* targets and guides expectant parents and parents of 0–2 year olds, serves as a reliable source of evidence-based information for midwives and public health nurses at maternal and child healthcare (MCH) centres, and offers pedagogical tools for early childhood education and care (ECEC) staff. The aim of this study is to implement *Nutrition Now* at scale and evaluate the impact of different sets of multifaceted implementation strategies on implementation outcomes.

**Methods:**

A quasi-experimental design with three study arms will be used, providing either low, medium or high implementation support, when rolled out in 50 municipalities in 2 counties in Norway. *Nutrition Now* will be implemented in MCH and ECEC settings and made available to expectant parents and parents of 0–2 year olds through social media and MCH. The implementation support builds on strategies described in the Expert Recommendations for Implementing Change (ERIC) implementation framework and is informed by dialogues with stakeholders. Impact of the different degree of implementation support will be assessed by examining reach, adoption, fidelity, and sustainability using usage data generated from the *Nutrition Now resource*, publicly available municipal data and qualitative interviews with MCH and ECEC staff.

**Discussion:**

*Nutrition Now* Phase 2 will break new ground by scaling up successively delivered and complementary dietary interventions in the first 1,000 days of life in a real-life context. The project also seeks to identify what level of implementation support is most effective when implementing digital, scalable, evidence-based early-life nutrition interventions in community settings. The project will inform implementation research and provide knowledge about effective implementation strategies to be used in a national scale-up of *Nutrition Now*.

**Trial registration:**

The study is registered prospectively (submitted 14/06/2022, registration date: 19/06/2022) in the International Standard Randomised Controlled Trial Number registry (ISRCTN): reg. Number: ISRCTN10694967, https://doi.org/10.1186/ISRCTN10694967.

## 1 Introduction

There are long-term benefits of early interventions and commitment to early childhood development, such as improving health, human capital and wellbeing across the lifecourse (1). A healthy diet during the first years of life is crucial for healthy growth, cognitive development, learning, current and future health, and well-being (2–6). Richter et al. have argued that the physical development of young children has been neglected, pointing at the need for scaled up interventions focusing on health and nutrition *during early childhood* (1). Since 2008 several Lancet Series on maternal and child nutrition have called for a movement for *Scaling up Nutrition* (7). However, few successful scientific “scale up studies” of nutritional interventions have been conducted (8, 9). The Australian INFANT study is an exception, which provides guidance on nutrition through interventions targeting parents of infants and toddlers through the health care system (10).

Scale-up and implementation are closely intertwined. Implementation is the process of integrating an intervention into practice within a particular setting (11, 12). Scale-up is “the process by which health interventions shown to be efficacious on a small scale and/or under controlled conditions are expanded under real world conditions into broader policy or practice” (13). Richter et al. identified crucial elements of the pathway to full-scale-up of interventions in early life (1). These include political prioritisation, creation of supportive policy environments, and the use of existing delivery systems to build further efforts and affordability (1). Further, Richter et al. identified the health sector and specifically the nutritional part as an important entry point for scale-up as there is already a system developed, reaching a large proportion of the target population.

Responding to the numerous calls for scaling up interventions early in life and the suggestions of Richter et al. (1), we have established the *Nutrition Now* project. In Phase 1 (14), we combined four efficacious dietary digital interventions, targeting the first 1,000 days, into a single e-learning resource (the *Nutrition Now resource*). This resource takes a lifecourse approach, targeting nutrition during sensitive dietary transitions, such as the initiation and maintenance of breastfeeding, the introduction of complementary foods, and the transition to early childhood education and care (ECEC) where meals are eaten outside the home environment and shared with peers (14).

In Norway, municipalities and counties are responsible for providing sound public health advice on diet and nutrition (15), however several municipalities do not have the workforce or the time to do so. According to our previous findings (16–18), maternal and child healthcare (MCH) nurses and midwives, who engage with over 95% of parents (19), report that providing guidance to parents on child diet and nutritional issues is part of their daily routine (20). They also report that they lack formal training in this area and thus often rely on their own experience (20), in addition to utilising information provided by national authorities, to guide parents. Swedish researchers have described similar findings, and state that MCH are in a key position to provide guidance, but often lack resources and time (21). ECEC staff are also strategically important in a public health perspective. In Norway the ECEC attendance in the age group 1-5-years is high (92%) (22). ECEC staff are responsible for the overall care of children while their parents work or study. Many children eat three meals a day in the ECEC setting (23), highlighting the importance of what is served and how food and meal settings are dealt with. The *Nutrition Now resource* comprising four digital diet interventions (14), offers a low-cost complementary tool for public health work in municipalities, and if successfully adapted when scaled-up, has the potential to improve population diet and health and respond to the need for tools and improved skills in childcare and ECECs. The *Nutrition Now resource* has a multisectoral approach and is directed towards parents and ECEC staff and includes elements relevant for MCH centres as well as those in the overarching municipality setting. These users are provided with research-based knowledge and skills to support children to acquire dietary habits that promote, their future health and development.

During the first phase of this project, the *Nutrition Now resource* was implemented in one municipality in Norway through municipal services for MCH care (age: 0–2 years) and ECECs for children aged 1–3 years. In the second phase of the *Nutrition Now* project, we aim to scale up the implementation of the *Nutrition Now resource* to county level, paving the way for potential national implementation. Within implementation research it has been suggested that implementation requires iterative support to enable addressing barriers as they appear (24); however, such strategies are resource intense. Currently, there is limited understanding of the amount of support required for successful implementation of an intervention, a key factor in considerations for scaling up. Further, to the best of our knowledge there have been no studies evaluating how different levels of implementation support in early life diet interventions impact implementation outcomes. Such studies are important to identify what level of implementation support health authorities need to provide for stakeholders to adopt new guidelines or interventions (25). In this study, we therefore aim to evaluate the effects of different sets of multifaceted implementation strategies/ levels of implementation support by introducing three levels (low, medium, and high support) when scaling up the *Nutrition Now resource* in 50 municipalities in Norway.

## 2 Methods

### 2.1 Study design

A quasi-experimental design consisting of three study arms will be used, two of which will be intervention arms and one will be the control arm. To ensure adherence to reporting standards, we followed the Standards for Reporting Implementation Studies (26).

**Figure 1:**
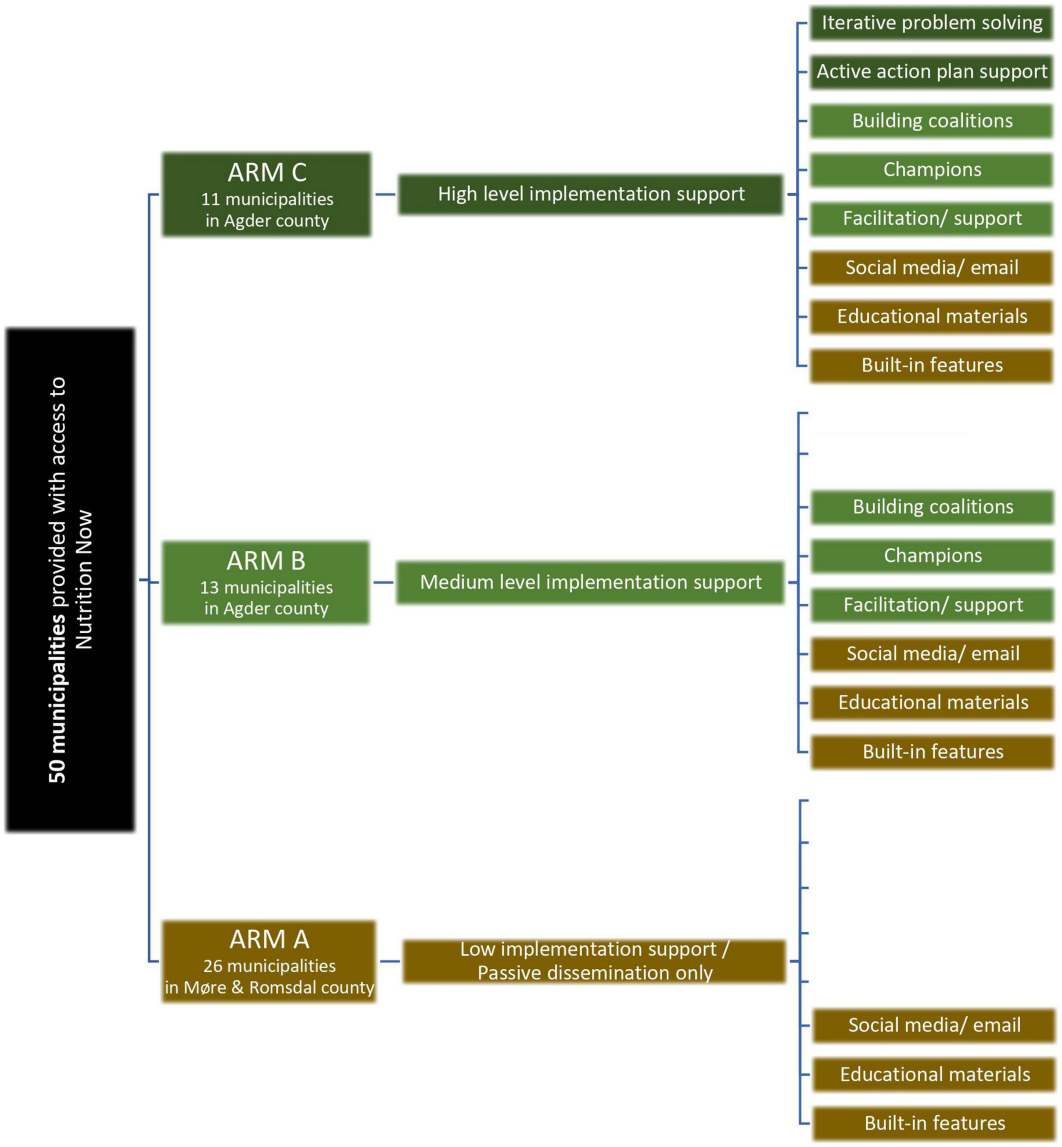
Design of the implementation study showing the three study arms. All study arms are provided with access to the *Nutrition Now resource*, whereas the level and type of implementation support varies between the study arms.

A total of 50 municipalities situated in two counties in Norway will be given access to the digital *Nutrition Now resource* (Figure 1). In the three different study arms, different implementation support regimes (sets of multifaceted implementation strategies) will be provided, ranging from low intervention support/no active support (study arm A) in the control county of Møre & Romsdal, to medium intervention support (study arm B) and high intervention support (study arm C) both in the county of Agder. Study arm A which serves as the control county will continue as normal and receive *Nutrition Now* through passive dissemination only.

### 2.2 Setting

The setting for this study is Norway, a country with 5.4 million residents, with many living outside major cities. The country has a strong tradition of local governance and decentralisation, also notably in its municipal systems and ECEC (27). Both the intervention and control counties are coastal counties and of similar size but situated in different regions of Norway (south and west). In Table 1, a comparison of the socio-demographics in the counties is shown. They have broadly comparable demographics and are comparable with respect to population size (310,000 vs. 270,000 inhabitants) (30), number of births per year (2,961 vs. 2,483) (31), and the number of ECEC centres (337 vs. 264) (32), among other factors.

**Table 1:** Comparability of control county (arm A) and intervention county (arm B and C) in Phase 2 of the *Nutrition Now* project (data from 2019 to 2023).

Characteristics ^a^	Control (C)	Intervention (I)	Difference (C-I)
Population size (2023) ^a^	268,365	311,134	−42,769
Annual number of births (mean for the years 2020–22) ^a^	2,483	2,961	−478
Population density (persons per km2) (2023) ^a^	19	21	−2
Immigrants and Norwegian-born to immigrant parents, % (2023) ^a^	15	17	−2
Higher educational level, people over 16y (1y+), % (2022) ^a^	31	33	−2
Higher educational level (long), people over 16y (4y+), % (2022) ^a^	7	9	−2
Median income per family after taxes in NOK (2021) ^a^	577,000	554,000	23,000
Persons per family/household (2023) ^a^	2	2	0
Children of single parents, % (2021) ^b^	13	16	−2
Children living in sustained low-income households, % (2019–21) ^b^	9	14	−5
Number of MCH centres (2023) ^c^	37	47	−10
Number of ECEC (2022) ^a^	264	337	−73
Private ECEC, % (2022) ^a^	47	64	−17
Children 1–2 years in ECEC, % (2022) ^a^	94	84	10

^a^Data from Statistics Norway (28). ^b^Data from the Norwegian Institute of Public Health (29). ^c^Data from the official homepages of the 50 municipalities in the counties of Møre & Romsdal and Agder.

### 2.3 Study participant groups and recruitment

All expectant parents and parents of 0–2 year olds living in the counties of Agder and Møre & Romsdal are eligible for this study. Further, all MCH leaders, MCH nurses and midwives, ECEC leaders, staff in participating ECECs, within all study arms (A, B or C) are eligible to participate. Other relevant municipality staff working within public health in municipalities within the study arms are also eligible.

**Figure 2:**
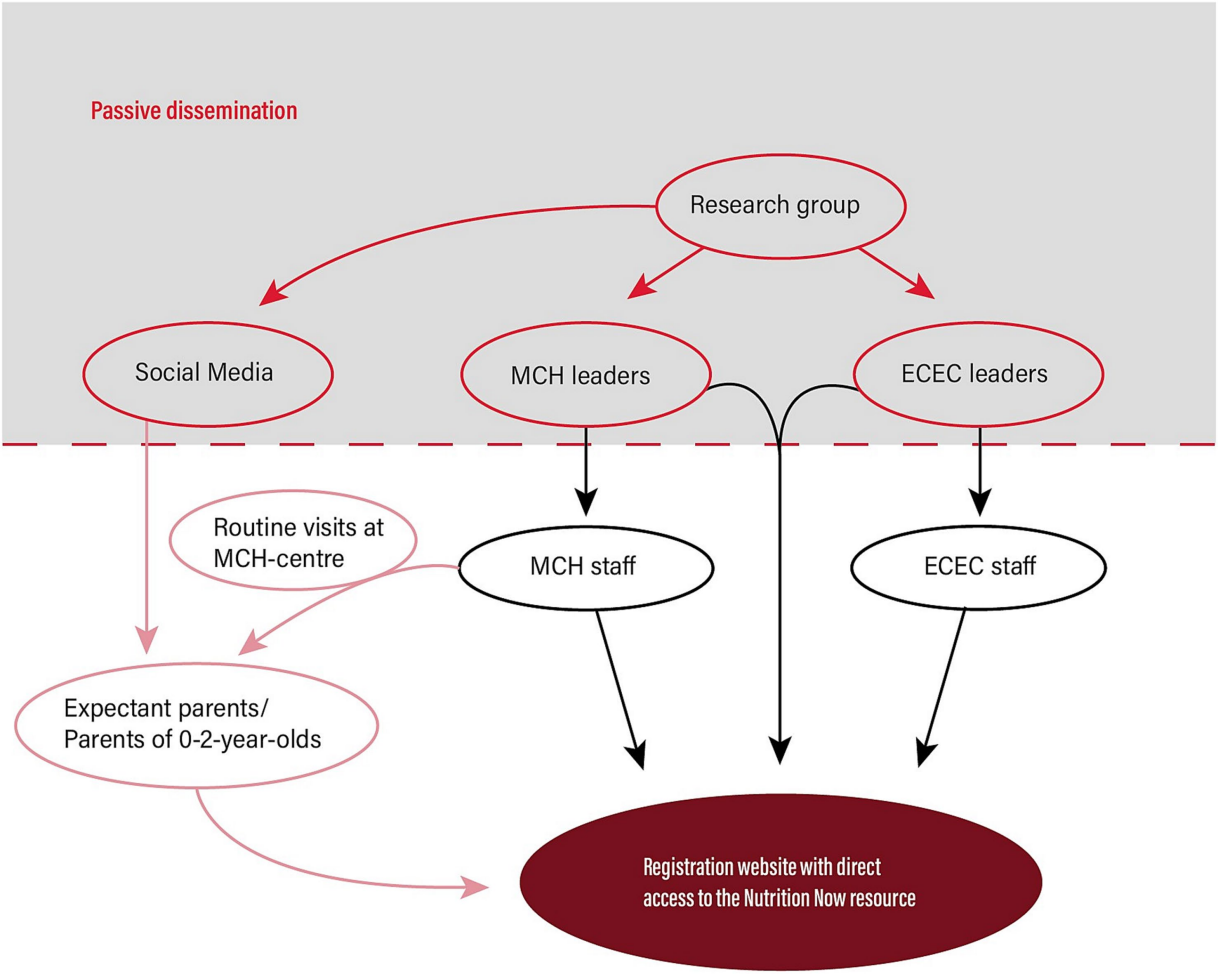
Overview of potential recruitment routes for Phase 2 in the *Nutrition Now* project.

An overview of potential recruitment routes for the participant groups Phase 2 in the *Nutrition Now* project is illustrated in Figure 2. Recruitment of participants will follow the same procedures in all three study arms and will start in the autumn 2023. Email invitations containing information about *Nutrition Now* Phase 2 and instructions on how to use the resource will be sent at the same time to ***MCH leaders*** and ***ECEC leaders*** in all three study arms. Additionally, we will publish social media posts, targeting ***expectant parents*** and ***parents of children aged 0–2 years**,* and linking to the *Nutrition Now resource,* which will be equally available across all three study arms. In study arm A, where low implementation support will be provided, participants will only gain access through passive dissemination. Disseminating the email and using social media channels will constitute the sole actions undertaken by the research group in study arm A. ***Expectant parents*** and ***parents of children aged 0–2 years*** may also be recommended to use the *Nutrition Now resource* by MCH nurses and midwives in all three study arms at routine visits to the MCH centre. All routes lead directly to the *Nutrition Now resource*, where they can sign up and provide digital consent. We will draw on our previous successful experience in recruiting participants using this approach (33, 34).

In all study arms, ***MCH and ECEC leaders*** within the municipalities will have the autonomy to determine the starting date for *Nutrition Now*. In study arms B and C, MCH leaders and ECEC leaders will consent on behalf of their staff and will subsequently be asked to recruit champions (contact persons/ team leaders) and other staff in their unit by forwarding digital invitations to participate in the study. In contrast, MCH leaders and ECEC leaders in arm A municipalities are informed that they have the option to share the *Nutrition Now resource* with other staff, but will independently decide how to implement the resource within their unit. ***Other municipality stakeholders*** (public health workers, leaders) and interested others, residing in the municipalities included in the three study arms, can also access the *Nutrition Now resource* by consenting digitally and creating a login profile. No specific actions will be taken to recruit these stakeholders.

### 2.4 The *Nutrition Now resource*

The *Nutrition Now resource* comprises four efficacious e-learning interventions (34–38), offered through a website targeting different early life transitions, from pregnancy to 2 years of age. It targets and guides expectant parents and parents of 0–2 year olds both directly and via municipal MCH and ECEC services. *Nutrition Now* also serves as a reliable source of evidence-based information for midwives and public health nurses at MCH services and offers pedagogical tools for ECEC staff.

Details, including theoretical underpinning of the interventions that the *Nutrition Now resource* comprises, are provided in protocol for Phase 1 of the project (14). The original interventions’ content and structure were modified and adapted to fit the context and user needs in the relevant settings for Phase 1 (family-, MCH centre- and ECEC- settings). The *Nutrition Now resource* is available in English and Arabic, in addition to Norwegian. The section for ECEC-setting is available only in Norwegian. The *Nutrition Now resource* covers several topics presented as core components, addressing the importance of *(1) diet early in life, (2) breastfeeding, (3) parental role in food provision and shaping a child’s diet, (4) responsive feeding, (5) shared meals, (6) knowledge and experience of food preparation, (7) sensory play involving vegetables* and *(8) ECEC collaboration with parents* (14). These topics are presented through short videos, graphics, images and easy-to-read text formats.

In Phase 2, based on insights from Phase 1, we have modified both the participant login process for the website and the way participants receive newsletters. These adjustments have automated the system, allowing us to evaluate resource usage and the time spent on it across all three study arms. We have also refined the menus and content in the ECEC section of the *Nutrition Now resource* based on insights from Phase 1, such as placing a greater emphasis on parent collaboration and adding more menu options. The content in the section of the resource designed for expectant parents and parents remains unchanged. However, we have supplemented the resource by providing the MCH with a specific website to their needs.

### 2.5 Project procedures in implementing the *Nutrition Now resource*

#### 2.5.1 Implementation strategies

The three different levels of implementation support in Phase 2 of *Nutrition Now* (low, medium, high) represent three sets of multifaceted and multilevel implementation strategies, targeting a range of stakeholders: municipal authorities, MCH staff, ECEC staff, expectant parents, and parents of 0–2 year olds in several settings. For the duration of the project period, we have employed a full-time implementation officer affiliated with the county authority of Agder. The officer’s role is to ensure that implementation support is executed as planned for the participating municipalities in arm B (medium) and C (high), all of which are in Agder.

**Figure 3:**
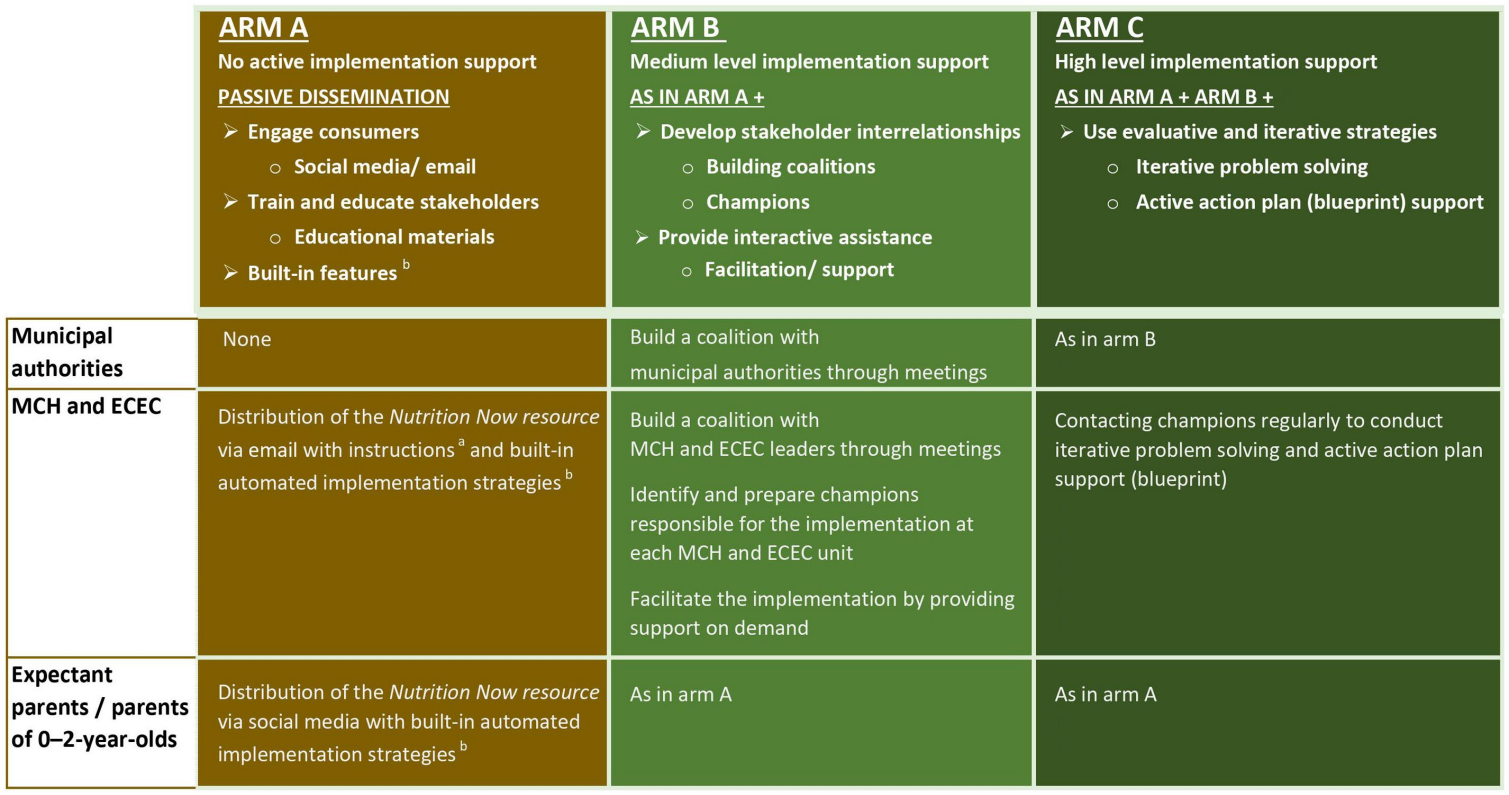
Selected sets of multifaceted and multilevel implementation strategies for the different study arms of Phase 2 of *Nutrition Now.* ^a^Educational materials refer to a broad category of resources provided to all study arms and includes everything from written information and videos describing the resource and how it has been intended being used, to guidance on how to make a tailored action plan (blueprint) at each MCH unit. ^b^In the *Nutrition Now resource* several implementation strategies are automated. These include email reminders to users with new and relevant content. In addition, the resource builds on and has incorporated previous implementation support from Phase 1. These strategies are described in detail in the *Nutrition Now* Phase 1 protocol (14).

The selected sets of implementation strategies for the different arms of Phase 2 targeting different stakeholders are shown in Figure 3, with additional details shown in Supplementary Table S1. Selected discrete strategies that make up the sets of implementation strategies in Phase 2 are automated and built into the *Nutrition Now resource.* Examples of such components are scheduled reminders and language customization. These automated strategies, incorporated into the *Nutrition Now resource,* have previously been described in detail in the *Nutrition Now* Phase 1 protocol (14).

##### 2.5.1.1 Selection of implementation strategies

Given the complexities and diversity in evidence-based interventions and their contexts, it is recommended to select and tailor strategies to fit each specific change effort (39). In Phase 1 of *Nutrition Now,* we observed and gathered information on the implementation process as an initial step to select suitable implementation strategies for Phase 2 of *Nutrition Now* (14). Leveraging this groundwork, we identified strategies to overcome barriers and building on the opportunities for implementation and sustainment in the MCH and ECEC settings in particular. Insights were also drawn from qualitative interviews with stakeholders during Phase 1 (unpublished). Through iterative discussions, our research team has identified and selected the strategies we deem most feasible and effective, tailored to the Phase 2 context. Additionally, in the *Nutrition Now* Phase 2, all the selected implementation strategies align with those deemed feasible and important according to the ERIC framework (Expert Recommendations for Implementing Change) (21). An overview of the implementation strategies selected for Phase 2 is provided in Figures 1, 3, while their operationalization according to the ERIC framework and justification are presented in Supplementary Table S1.

##### 2.5.1.2 Implementation strategies targeting the municipal authority level

Implementation strategies targeting the authority level in municipalities will be exclusively provided to municipalities in study arms B and C, receiving medium and high implementation support, respectively.

The support includes developing stakeholder interrelationships by informing and engaging administrative leaders and stakeholders at the municipal level, as engagement of these groups is considered important for the municipality to take ownership of the project and for the MCH centres and ECEC centres to perceive *Nutrition Now* as an integral part of the municipal strategy. Therefore, we will arrange meetings and inform leaders in the municipality at various levels, including the Chief Executive, heads of educational sector and health sector, MCH leaders and ECEC leaders. Moreover, based on the experiences from Phase 1, we deem a close collaboration with the municipal Public Health Coordinator as central for a successful implementation. Each municipality in study arms B and C will assign one contact person for *Nutrition Now*, and this person should preferably be the Public Health Coordinator.

##### 2.5.1.3 Implementation strategies targeting MCH staff

All MCH units, including those with ***low implementation support*** in study arm A, will be provided with the *Nutrition Now resource* and educational materials, including instructions on how to use the resource and engage parents. The distributed material includes written information, a video, and an exclusive webpage with additional resources for the MCH staff, as well as guidance on how to incorporate the *Nutrition Now resource* into the daily practice of the MCH units.

For the municipalities receiving ***medium implementation support*** in study arm B, we will add interrelationships and interactive assistance. Through the heads of the municipal health sector, MCH leaders are invited to partake in meetings with members from the research team, either in physical or digital format. This is done so that the use of *Nutrition Now* is rooted in the work of the MCH. Further, we will actively ask MCH leaders to designate a staff member who will serve as a contact person. This individual, referred to as a champion in accordance with the ERIC framework (40), will be responsible for initiating the use of the resource within the MCH and integrating it into routine operations.

In municipalities with ***high implementation support*** in study arm C, we will add evaluative and iterative strategies in the form of regular contact (primarily calls) with the MCH units from a staff member in the *Nutrition Now* team. This support will be provided for the first 8 months after initiating *Nutrition Now*. There will be monthly contact for the first 2 months, moving to bi-monthly thereafter. This approach aims to provide support for problem solving and action plan (blueprint), to improve the implementation process through iterative improvement cycles.

##### 2.5.1.4 Implementation strategies targeting ECEC staff

ECEC units in all three study arms, including those with ***low implementation support*** in study arm A, will receive access to the *Nutrition Now resource* and educational materials distributed through passive dissemination, and will also have access to built-in automated implementation strategies once they have accessed the resource.

For the ***medium implementation support*** municipalities, we will in addition establish a working relationship with ECEC leaders. This includes anchoring of *Nutrition Now* through meetings comprising ECEC leaders and research team members, either in physical or digital format, endorsed by the heads of the municipal educational sector. Moreover, the ECEC leaders will be asked to identify champions (team leaders) in the ECEC to be responsible for implementing *Nutrition Now* in the ECEC in line with the instructions provided. The champions may contact the research group if they have questions and need support.

In the ***high implementation support*** municipalities, in addition to all the measures in the medium support localities, the champions will be actively contacted regularly for the first 8 months after initiating *Nutrition Now*, monthly for the first 2 months and then bi-monthly, to support and improve the implementation process through iterative improvement cycles.

##### 2.5.1.5 Implementation strategies targeting expectant parents and parents of 0–2 year olds

Social media will be used to disseminate the resource directly to expectant parents and parents of 0–2 year olds in all three study arms, including those with ***low implementation support*** in study arm A. Participating MCH units in municipalities in all study arms serve as an additional dissemination channel, distributing information about the *Nutrition Now resource* to expectant parents and parents of 0–2 year olds during routine visits.

In the study arms with ***medium*** and ***high implementation support***, the support targeting MCH staff described above is intended to indirectly influence parental reach and use of *Nutrition Now*.

### 2.6 Outcomes

The current study is an implementation study, and while we have primarily focused on implementation outcomes, we are also incorporating a cost analysis. The selection of specific implementation outcomes was informed by the widely accepted RE-AIM framework (41), ensuring alignment with established practices in implementation research. An overview of the implementation outcomes selected for Phase 2 in *Nutrition Now*, how they were defined, target groups, and data sources is shown in Table 2.

**Table 2:** Overview of planned implementation outcomes.

Outcome	Question and definition	Target group(s)	Data source(s)
Quantitative implementation outcomes			
Reach	What proportion of *Nutrition Now*’s target population receive it (created an account to use the resource)? Number of registered users of the *Nutrition-Now resource*, divided by total number of eligible individuals in each municipality: Calculated for each calendar quarter from study start date to completion at 24 months.	Stratified by: (a) Expectant parents/ parents of 0–2 year olds (b) MCH staff (c) ECEC staff	i. *Nutrition Now resource*: number of users ii. Statistics Norway: Quarterly data on number of eligible expectant parents/ parents of 0–2 year olds in each municipality iii. The Norwegian Directorate for Education and Training: Number of MCH and ECEC staff in each municipality
Adoption	What is the proportion of settings that are willing to initiate *Nutrition Now*? How is the representativeness of these settings that are willing to initiate *Nutrition Now*? (a) Number of leaders of MCH and ECEC leaders who are willing to initiate the *Nutrition Now* at their workplace (created a profile), divided by total number of eligible leaders in those groups (received email in Phase 2). (b) Comparisons of representativeness are municipal population size and density, and municipal budget (*per capita*); for ECECs also the proportion private vs. public ECECs and the proportion of children in ECECs. Calculated for each calendar quarter from study start date to completion at 24 months.	(a) MCH leaders (b) ECEC leaders	i. *Nutrition Now resource*: which MCH leaders and ECEC leaders have created a profile ii. The Norwegian Directorate for Education and Training: info on ECECs and contact info for ECEC leaders iii. Municipal statistics: info on MCH units, contact info for MCH leaders, municipal budget size iv. Statistics Norway: Financial key figures for municipalities; population size and density; ECEC attendance
Maintenance (sustainability)	Is the *Nutrition Now resource* is still being used at ≥6 month post study? How much is the resource accessed? How much time is spent using the resource? (a) Total number of logins and (b) Total time logged in, aggregated and standardised by the number of users in each municipality. Calculated for each calendar quarter from study start date to completion at 24 months.	Pooled over all *Nutrition Now* registered users (expectant parents/ parents of 0–2 year olds/ MCH staff/ ECEC staff/ interested others)	i. *Nutrition Now resource*: Log-ins; time spent in resource and number of registered users
Qualitative implementation outcomes			
Implementation (fidelity)	How did MCH and ECEC staff use the *Nutrition Now resource*? What adaptations were made to the intervention during the study?	(a) MCH staff (b) ECEC staff	Qualitative digital interviews ≥6 month post study in all three arms
Maintenance (sustainability)	Has *Nutrition Now* been institutionalised? How was it done? Has *Nutrition Now* been adapted for long-term use? If yes, which elements were retained?	(a) MCH staff (b) ECEC staff	Qualitative digital interviews ≥6 month post study in all three arms

### 2.7 Data collection

Both quantitative and qualitative data will be collected to evaluate the implementation outcomes and we will conduct cost analysis in all three study arms. This will include data from the *Nutrition Now resource* and official municipality statistics (e.g., number of births within a quarter in a municipality), as well as qualitative interviews for implementation outcomes and brief survey-based interviews for cost analysis.

To assess ***reach*,** usage data from individual users of the *Nutrition Now resource* will be used in addition to openly available official municipality statistics. Reach is defined as the proportion of eligible individuals who engage in *Nutrition Now*. Reach for the following groups will be assessed: (i) *expectant parents, parents of 0–2 year olds*, (ii) *MCH staff*, and (iii) *ECEC staff*. The reach within each of these groups will be quantified by dividing the number of users who have created an individual profile on the *Nutrition Now resource* within a group by the total number of eligible individuals within that specific group. To study time trends, we will analyse data on reach at quarterly intervals from the start of the study, which begins when emails are distributed to the services and parents are invited through social media. The final assessment of total use will occur 24 months after study start. The denominator at each time point will therefore correspond to the numbers of users within the different groups at that specific quarter, year, and for the most recent year in cases where data are only updated annually. The data sources for openly available statistics will be as follows: (i) Statistics Norway providing population counts, including number of births and children between 0 and 2 years of age within each municipality in Norway quarterly (42), for feasibility reasons the denominator will be calculated using the number of children between 0–2 year olds in addition to the mean quarterly number of births based on data from the last 12 months (as an estimate of number of expectant parents); (ii) The Norwegian Directorate for Education and Training providing numbers on total number of ECEC staff, reported for each year, in the municipality (multiply ECEC staff per child with the number of children attending ECEC) (32); (iii) Statistics Norway providing numbers on MCH staff, reported for each year (43).

***Adoption*** will be assessed for ECEC and MCH settings by using data on the proportion and representativeness of ECEC and MCH leaders who are willing to initiate *Nutrition Now* in their unit. Specifically, the proportion of eligible ECEC and MCH leaders who acted upon the email invitation in which they are given access to the resource (by logging in and creating an account), divided by total number of eligible leaders in that group. Comparisons of representativeness of ECEC and MCH units will be made on the basis of data from Statistics Norway on municipal size and density (44), and municipal budget (*per capita*) (44); and for ECECs on proportion of private vs. public ECECs (32), and the proportion of children in ECECs in the municipality (44).

***Maintenance (sustainability)*** will be assessed quantitatively using data on usage of the *Nutrition Now resource* after ≥6-months post provision of implementation support in the study. The total use of the *Nutrition Now resource* across the different study arms will be assessed by calculating the total number of log-ins and total usage time, which will be divided by the number of unique users in each study arm, respectively. In the same way as for reach, we will analyse data on reach at quarterly intervals from study start and 24 months onwards to evaluate time trends. The denominator at each time point will correspond to the numbers of users in that specific quarter, year. Data for the denominator will be extracted from Statistics Norway, who provide updated population counts within each municipality in Norway quarterly (42).

Sustainability will also be assessed using qualitative methods. Digital individual interviews with a randomly selected sample of MCH and ECEC staff will be conducted in all three study arms, including both champions and other staff. Specifically, for the ECECs, this data collection will be done around 6 months after the end date for the first cycle (starting date) of using *Nutrition Now*, e.g., if started in November 2023, following the program cycle for 6 months, until May 2024, then they would be interviewed around November 2024. For the MCHs, this will be a year after the MCH leader first created a user in the *Nutrition Now resource.*

***Implementation (fidelity)*** will be assessed using qualitative methods, as described for sustainability.

***Cost data*** will be collected to enable cost analysis. Costs related to resources used in the implementation of the *Nutrition Now* Phase 2 will be collected from participating municipalities, MCH centres, and ECECs. Estimated resource use will include the comparative costs or saving of the *Nutrition Now* and changes in infrastructure, use and maintenance. Staff training costs will be included. Time (in minutes) spent by staff on the implementation of *Nutrition Now* Phase 2 in MCH centres and ECECs will be collected using brief survey-based interviews.

### 2.8 Analysis

***Quantitative*** outcomes are reach, adoption and sustainability (see Table 2 for definitions). The main comparisons will be between groups A (low support) and B (medium support), and groups B and C (high support). The former will provide an estimate of the effect of medium implementation support, the latter will estimate the effect of additional iterative implementation support. Adjustment for multiple comparisons will be conducted.

Since the outcomes may vary over the implementation period, which may also depend on the level of implementation support received, we will assess the trajectories of each outcome using the quarterly repeated measures from the study start date. General linear models and generalised estimating equations (for analysis involving repeated measures) will be used to estimate the intervention effects and to estimate the marginal means by groups. We will also explore the association between adoption and characteristics of the municipality (size, density, budget) and ECEC (private/ public ownership and attendance).

***Qualitative*** outcomes are fidelity and sustainability (see Table 2 for definitions). Qualitative interview data will be collected and subsequently transcribed verbatim and coded deductively informed by the Consolidated Framework for Implementation Research framework (CFIR) (45), and inductively to capture issues within and outside of this framework. We will use these findings to inform future scale-up at a national level.

***Cost analysis*** comparing different levels of implementation supports offered to three study arms will also be conducted.

### 2.9 Randomisation of municipalities

The Research Randomizer (Version 4.0) (46) has been used to randomise the municipalities in the study. For practical reasons, Agder county (with 24/25 municipalities included in study) was chosen to receive implementation support, whereas Møre & Romsdal county (with 26/26 municipalities included in study) was selected to serve as the control county. The one municipality in Agder county excluded from the study was the site for a hybrid type 1/Phase 1 *Nutrition Now* study.

Before randomisation, the 24 municipalities in Agder were grouped by population size and divided into four randomisation blocks. Block 1 had 8 municipalities with <2,000 inhabitants; Block 2 had 8 with 2,000-8,999; Block 3 had 4 with 9,000-11,999; and Block 4 had 4, with one having ~116,000 inhabitants and three with 15,000-25,000. Blocks 1–3 were randomised with half of the municipalities receiving medium and others high intervention support. Block 4, due to size disparities, were divided into two groups: one comprising the three least populated municipalities (in block 4) and the other comprising the single most populated municipality, with one group getting high and the other medium support after randomisation.

The randomisation is partially blinded. Specifically, the municipalities involved in the study are not informed about whether they are receiving high, medium, or low support.

### 2.10 Sample size

To ensure the study is adequately powered to detect significant differences between study arms, we used the implementation outcome ***reach*** – defined as the proportion of eligible individuals who receive *Nutrition Now*. For detecting as little as a 4% difference in reach between the control arm (arm A, low level support) and arm B (medium level support), given that the reach in the control arm lies between 10 and 50%, our power exceeds 96%. When comparing arm B (medium level support) with arm C (high level support), assuming any value for reach in arm B, we maintain a power of at least 93% to detect differences of 4% between the groups.

### 2.11 Data management

A data management plan (DMP) for the project has been developed by following the template provided by the Norwegian Agency for Shared Services in Education and Research (Sikt) (47). The DMP is an ongoing document throughout the project period and the Sikt DMP template is recommended by and in accordance with Norwegian Research Council’s (NRC) requirements and guidelines. We will share anonymized data in the UiA data repository Dataverse. This will be done no later than upon acceptance for publication of the main findings from the final dataset. We will retain our data for 5 years after data collection has stopped. Hence, our data will be made open access in line with the NRC’s guidelines, prior to 3 years after the completion of the study. Standard meta-information about the data will be uploaded.

Quantitative participant data in this study will be collected using usage data collected through the *Nutrition Now resource*, collected and stored in Matomo (48), a web analytics application. Interviews will be recorded and transcribed, and stored in a secure server, the Service for Sensitive Data (TSD, in Norwegian, “Tjeneste for Sensitive Data”). TSD is constructed for storing and processing data in agreement with the Norwegian Personal Data Act and Health Research Act (49).

## 3 Discussion

The fundamental importance of diet early in life for long-term health and wellbeing is indisputable (5), and successful ways of improving diet early in life have been documented in randomised controlled trials (34–38, 50). Still, few studies have implemented efficacious dietary interventions at scale (1, 10, 50). The *Nutrition Now* Phase 2 will break new ground by scaling up a set of dietary interventions in the first 1,000 days of life in a real-life context (1). Lifecourse research tends to be skewed towards epidemiology rather than implementation, and commonly targets single transitional phases (51). Our project addresses these scientific shortcomings by implementing and evaluating evidence-based e-learning resources across multiple transitions early in life in a real-world community setting. Further, *Nutrition Now* is also responding to the call for multisectoral approaches, as it takes advantage of several existing settings across sectors in municipal care (MCH and ECEC) to secure universal reach (1). *Nutrition Now* is also one of the first implementation studies to evaluate the level of implementation support required for individuals and sectors to use a health-promoting digital resource (10).

By gaining knowledge of what is needed in terms of implementation support to maximise use of the digital resource, we will be able to make informed choices about how to implement at national level, thereby contributing significantly to evidence-based health services in municipalities across Norway.

There are several potential challenges this project may face. One potential limitation is that our target groups are not well enough positioned to implement in the short time frame of this study. Further, the study aims to evaluate how different levels of implementation support (low vs. medium vs. high) impact the use of the digital resource. A true limitation might be that the differences in input between the three implementation supporting levels may be too small, and that we will not be able to detect differences in *Nutrition Now* use. We have added the strategy of iterative re-examination of implementation processes to the high support arm. This is because this strategy seems to have been most useful and appreciated in Phase 1 (data not published), however it is also one that is time consuming and resource intense for the research team and will need to be well justified if it is to be used at a national level. The difference from low support to medium is more evident since medium support includes both developing stakeholder interrelationships and facilitation, which is not included in the low support group.

A major strength of this study is the established efficacy of the resource (34–36, 38). Another strength is that the digital resource and the strategies have been co-created with relevant stakeholders in one municipality. The fact that Phase 2, including the chosen implementation strategies, build on experiences from Phase 1 will help address challenges along the way.

## 4 Ethics and dissemination

The project has received approval from the Regional Ethics Committee (REC), the University of Agder’s Faculty Ethical Committee (FEC), and the Norwegian Data Protection Service (NSD; ID numbers: REC: 322480, NSD: 847590). Any necessary protocol changes will be submitted to the REC, the NSD, and the trial registry ISRCTN (ISRCTN10694967). Participation is voluntary, with participants needing to actively give digital consent. They are free to withdraw their consent at any time, without providing a reason. Participants agree to their data being used in ancillary studies. Personal information will be protected in TSD, and no individual data from this project will be disseminated. Only aggregate and anonymized data will be published. Study outcomes will be published in peer-reviewed journals and disseminated at scientific conferences. Furthermore, we will share findings with stakeholders via meetings and our project’s website. Main findings will also be communicated to the broader public through the media.

## Ethics statement

The studies involving humans were approved by the Regional Ethics Committee (REC), the University of Agder’s Faculty Ethical Committee (FEC), the Norwegian Data Protection Service (NSD). The studies were conducted in accordance with the local legislation and institutional requirements. The participants provided their written informed consent to participate in this study.

## Author contributions

ACM: Conceptualization, Funding acquisition, Methodology, Project administration, Writing – original draft, Writing – review & editing. FNV: Conceptualization, Funding acquisition, Project administration, Writing – review & editing. CH: Conceptualization, Writing – review & editing. SHH: Conceptualization, Writing – review & editing. AKW: Conceptualization, Methodology, Writing – original draft, Writing – review & editing. NGO: Conceptualization, Writing – review & editing. HL: Conceptualization, Writing – review & editing. TIE: Conceptualization, Writing – review & editing. WVD: Conceptualization, Writing – review & editing. TB: Conceptualization, Writing – review & editing. ENV: Conceptualization, Writing – review & editing. MKG: Conceptualization, Writing – review & editing. EG: Conceptualization, Writing – review & editing. CK: Conceptualization, Writing – review & editing. UTS: Conceptualization, Writing – review & editing. MHA: Conceptualization, Writing – review & editing. PL: Conceptualization, Writing – review & editing. KC: Conceptualization, Writing – review & editing. HR: Conceptualization, Writing – review & editing. MEB: Conceptualization, Writing – review & editing. ERH: Conceptualization, Funding acquisition, Project administration, Writing – review & editing. NCØ: Conceptualization, Funding acquisition, Project administration, Writing – original draft, Writing – review & editing.
