# Supplementary material for: Scaling up evidence-based digital early life nutrition interventions in a county setting: an implementation trial – protocol for Phase 2 of the Nutrition Now project
Source: Front Public Health. 2024 Jan 9;11:1326787. doi: 10.3389/fpubh.2023.1326787 (PMC10803599; doi:10.3389/fpubh.2023.1326787)
Supplement: Supplementary file 1 [file Table_1.DOCX]

Table S1: Implementation strategies targeting *Nutrition Now* implementation delivered through Municipal Authorities, Maternal and Child Health Care (MCH) and Early Childhood Education and Care (ECEC) in the different study arms.

| **Study arm** | **Category (1, 2)** | **Strategy (# denotes ERIC implementation strategy) (3)** | **The Actor**  ***(deliver strategy)*** | **The Action**  ***(specific action, steps or process)*** | **Action Target**  ***(affected by strategy)*** | **Temporality and Dose** | **Justification** |
| --- | --- | --- | --- | --- | --- | --- | --- |
| **Municipal Authorities** | | | | | | | |
| **Arm A** | **No active support** |  |  |  |  |  |  |
| **Arms B and C** | **Develop stakeholder interrelationships** | # 6 Build a coalition | Implementation researchers | Conduct meetings with stakeholders (leaders within public health, health care services and ECEC) | Stakeholders in municipality | Pre-implementation and during implementation | Ensure a good collaborative environment between stakeholders and research team at the municipality level |
| **MCH** | | | | | | | |
| **Arms A, B and C^1^** | Implementation strategies included in the *Nutrition Now resource* | | | | | |  |
| **Arms A, B and C** | **Train and educate stakeholders** | #29 Develop educational materials | Implementation researchers in collaboration with web designer | A short manual and a video for MCH nurses and midwives explaining how to convey the content and use of the *Nutrition Now resource* to parents.  An exclusive website for health care personnel on how to use the resource and educational materials. | MCH nurses and midwives | Pre-implementation | Need for instructional materials to make it easier for staff to convey the *Nutrition Now* *resource*.  Increase MCH staff’s knowledge about *Nutrition Now resource* content and project fundamentals |
|  |  | # 31 Distribute educational materials | Implementation researchers | Distribute educational material by e-mail (including link to website), including guidance on how to make a tailored action plan (blueprint) at each MCH unit | MCH leaders and champion | Pre-implementation | Provide educational materials to staff to facilitate the delivery of the intervention |
| **Arms B and C** | **Develop stakeholder interrelationships** | #6 Build a coalition | Implementation researchers | Inform MCH leaders by email and digital meetings. | MCH nurses and midwives | Pre-implementation | Ensure a good collaborative environment between stakeholders at the setting level |
|  |  | #35 Identify and prepare champions | MCH leaders | Select a champion at each MCH who is responsible for the implementation at the MCH level | Champion at MCH | Pre-implementation | Anchor and ensure the implementation at each MCH unit |
| **Arm C** | **Use evaluative and iterative strategies** | #23 Develop formal implementation Blueprint | MCH nurses and midwives | MCH staff work with an action plan tailored to their needs, supported by implementation researcher | MCH staff |  | Create ownership to *Nutrition Now* and assist in finding ways to use *Nutrition Now* in daily practice |
|  |  | #56 Purposefully reexamine the implementation | Implementation researcher | Contact champions regularly to improve implementation | MCH nurses and midwives | During the implementation process. Monthly first 2 months, then bimonthly | Optimize implementation strategies |
| **ECEC** | | | | | | | |
| **Arms A, B and C^1^** | Implementation strategies included in the *Nutrition Now resource* | | | | |  |  |
| **Arms A, B and C** | **Train and educate stakeholders** | # 29 Develop educational materials | Implementation researchers | Develop educational material | ECEC leaders and champion | Pre-implementation | Provide educational materials to staff to facilitate the delivery of the intervention |
|  |  | # 31 Distribute educational materials | Implementation researchers | Distribute educational material by e-mail | ECEC leaders and champion | Pre-implementation | Provide educational materials to staff to facilitate the delivery of the intervention |
| **Arms B and C** | **Develop stakeholder interrelationships** | # 35 Identify and prepare champions | ECEC leaders | Identify a champion at each ECEC and clarify understanding of the role as an implementation agent | Champion at ECEC | Pre-implementation | Anchor and ensure the implementation at each ECEC unit |
|  |  | # 6 Build a coalition | Implementation researchers | ECEC leaders attend information meetings | ECEC leaders | Pre-implementation | Ensure a positive collaborative environment and build stakeholder relationships |
| **Arms B and C** | **Provide interactive**  **assistance** | # 33 Facilitation | Implementation researcher | Supportive implementation officer available for problem solving | ECEC Staff, ECEC champions | Ongoing during implementation | Ensure a positive collaborative environment between ECEC stakeholders at the setting level |
| **Arm C** | **Use evaluative and iterative strategies** | # 56 Purposefully reexamine the implementation | Implementation researcher | Contact champions regularly to improve implementation | ECEC champions | During the implementation process. Monthly first 2 months, then bimonthly | Use the input from champions to ensure quality and improvement of implementation strategies |
| **Expectant parents and parents of 0-2 year olds** | | | | | | | |
| **Arms A, B and C** | **Engage consumers** | #69 Use (social) media | Implementation researchers | Use social media to reach parents and enable them to use the *Nutrition Now* | Expectant parents/ parents of 0-2-year-olds | Implementation start | Provide access to the target population and maximize the spread of the resource |
| **Arms A, B and C^1^** | Implementation strategies included in the *Nutrition Now resource* | | | | | | |

^1^ In the *Nutrition Now resource* several implementation strategies are built-in and automatic. These include email reminders to users with new and relevant content. In addition, the resource builds on and has incorporated previous implementation support from Phase 1. These strategies are described in detail in the *Nutrition Now* Phase 1 protocol (4).

^2^ Educational materials refer to a broad category of resources provided to all study arms and include everything from written information and videos describing the resource and how it has been intended to be used, to guidance on how to make a tailored action plan (blueprint).

1. Waltz TJ, Powell BJ, Matthieu MM, Damschroder LJ, Chinman MJ, Smith JL, et al. Use of concept mapping to characterize relationships among implementation strategies and assess their feasibility and importance: results from the Expert Recommendations for Implementing Change (ERIC) study. Implement Sci. 2015;10:109.

2. Proctor EK, Powell BJ, McMillen JC. Implementation strategies: recommendations for specifying and reporting. Implement Sci. 2013;8:139.

3. Powell BJ, Waltz TJ, Chinman MJ, Damschroder LJ, Smith JL, Matthieu MM, et al. A refined compilation of implementation strategies: results from the Expert Recommendations for Implementing Change (ERIC) project. Implement Sci. 2015;10:21.

4. Øverby NC, Hillesund ER, Helland SH, Helle C, Wills AK, Lamu AN, et al. Evaluating the effectiveness and implementation of evidence-based early-life nutrition interventions in a community setting a hybrid type 1 non-randomized trial - the Nutrition Now project protocol. Front Endocrinol (Lausanne). 2022;13:1071489.
